# Increased subsequent risk of mental disorders after experiencing stress-related disorders: correspondence

**DOI:** 10.1097/MS9.0000000000000295

**Published:** 2023-03-14

**Authors:** Dmytro I. Boiko, Anastasiia D. Shkodina

**Affiliations:** Departments of a Psychiatry, Narcology and Medical Psychology; b Neurological Diseases, Poltava State Medical University, Poltava, Ukraine

*Dear Editor*,

Stress-related mental disorders represent a major risk for people’s socioemotional and physical wellbeing. The association between post-traumatic stress disorder (PTSD) and a wide range of health disorders demonstrates how far-reaching the impacts of severe stress are. The main targets for therapy of PTSD are large criteria, such as intrusion, avoidance, negative alterations in cognitions and mood, increased arousal, and reactivity, while single symptoms may persist. Many researchers focus on the remitting course of PTSD or its chronicity. However, we assume that the persistence of individual post-traumatic symptoms after treatment is equally important.

Substantial evidence indicates connections between PTSD and a range of downstream physiological alterations that might come from this stress response dysregulation and lead to the beginning of illnesses. Dysregulation of the hypothalamic–pituitary–adrenal axis and the autonomic nervous system, for example, can result in sleep disorders, inflammation, and oxidative stress^1^.

The study on Vietnamese refugees shows that stress-related mental disorders have long-term effects on the body even decades later, possibly due to stress sensitization^2^. Stress sensitization can result in increased sleep reactivity and residual sleep disruption after standard PTSD treatments, while therapies for sleep disruption have been shown to reduce PTSD symptoms^3^. Stress-related rumination and fixation may exploit sleep regulatory mechanisms, therefore amplifying sleep reactivity’s pathogenicity. Sleep quality also reduced the association between anxiety sensitivity and PTSD symptoms^4^.

The probability of increasing sleep disorders as a result of the war in Ukraine has been discussed in previous studies, which can potentiate the manifestations of mood disorders and stress-related disorders^5^. Considering the two so-called ‘waves’ of Russian invasion of Ukraine in 2014 and 2022, we may expect that among this cohort, hypotheses concerning the long-term repercussions of stress-related disorders will be studied and will provide important findings for a better understanding of this field.

Traumatic experiences can have long-term impacts on a person’s brain structure and function, and it is hypothesized that these changes may lead to mental diseases such as mood disorders and psychosis. There was a high comorbidity between PTSD, schizophrenia, and bipolar disorder. Thus, in different research, the average prevalence of PTSD in people with schizophrenia ranges from 12 to 29%, reaching 16% in people with bipolar disorder^6^.

Furthermore, while adjustment disorder is thought to be a more benign and short-term process than PTSD, it has been linked to long-term illnesses and a tendency to mental diseases, which can also be a result of neuroendocrine disturbance following traumatic exposure. Changes in the homeostasis as a result of a stress-related disorder are also significant, as they are reflected in the functioning of the entire body and reflect in the form of systemic consequences. A retrospective nationwide cohort study based on a Swedish registry found that not only PTSD patients but also people with other stress-related psychiatric disorders had a significantly increased relative risk of immune dysfunction, which is consistent with some biological evidence linking psychological stress and stressful events to various immune disorders^7^. Furthermore, neuroinflammation through proinflammatory cytokines is linked to neuroplasticity, neurogenesis, and gliogenesis, and it plays a role in the pathophysiology of mood disorders^8^.

Based on multiple studies, we emphasize isolated findings regarding the long-term implications of stress-related mental diseases, particularly alterations in the neurological, immunological, and endocrine systems’ homeostasis, as well as an increased risk of occurring other psychiatric disorders. Thus, despite substantial theoretical evidence, the mechanism and duration of alterations caused by stress-related disorders remain unexplained. Because the number of factors that can act as traumatic factors and lead to the development of stress-related disorders is increasing in the modern world, we should pay attention not only to the relief of acute symptoms of the disease but also to the evaluation of its prognosis and long-term consequences for improving global health.

## Ethical approval

Not applicable.

## Consent

Not applicable.

## Sources of funding

None.

## Conflicts of interest disclosure

Authors declare no conflict of interests.

## Provenance and peer review

Not commissioned, externally peer reviewed.
